# Understanding the mechanisms of green tea EGCG against amyloid β oligomer neurotoxicity through computational studies†

**DOI:** 10.1039/d4ra03343d

**Published:** 2024-07-16

**Authors:** Priscila Baltazar Gonçalves, Yraima Cordeiro, Ana Carolina Rennó Sodero

**Affiliations:** a Faculdade de Farmácia, Programa de Pós-Graduação em Ciências Farmacêuticas, Universidade Federal do Rio de Janeiro RJ 21941-902 Brazil priscilabaltazar@ufrj.br acrsodero@pharma.ufrj.br

## Abstract

Oligomeric species of amyloid β peptide (Aβ) are pivotal in Alzheimer's disease (AD) pathogenesis, making them valuable therapeutic targets. Currently, there is no cure or preventive therapy available for AD, with only a few therapeutics offering temporary alleviation of symptoms. Natural products (NPs) are now considered promising anti-amyloid agents. Green tea catechins have garnered considerable attention due to their ability to remodel the toxic amyloid β peptide oligomers (AβOs) into non-toxic assemblies. Nevertheless, the precise molecular mechanism underlying their effects on AβOs remains unclear. In this study, we employ a combination of binding site prediction, molecular docking, and dynamics simulations to gain mechanistic insights into the binding of the potent anti-amyloid epigallocatechin-3-gallate (EGCG) and the less effective catechin, epicatechin (EC), on the structure of pore-forming Aβ tetramers (PDB ID 6RHY). This recently elucidated structure represents AβO_(1–42)_ with two faces of the hydrophobic β-sheet core and hydrophilic edges. Our simulations revealed three potential druggable binding sites within the AβO: two in hydrophilic edges and one in the β-sheet core. Although both catechins bind *via* hydrogen bond (H-bond) and aromatic interactions to the three potential binding sites, EGCG interacted with key residues more efficiently than EC. We propose that EGCG may remodel AβOs preventing pore formation by binding to the hydrophilic edge binding sites. Additionally, EGCG interacts with key residues in the oligomer's β-sheet core binding site, crucial for fibrillar aggregation. A better understanding of how anti-amyloid compounds remodelling AβOs could be valuable for the development of new therapeutic strategies targeting Aβ in AD. Further experimental validation using point mutations involving key residues could be useful to define whether the establishment of these interactions is crucial for the EGCG remodelling effect.

## Abstract 2

EGCG and EC bind to the same sites on AβOs. However, EGCG forms H-bond and π-interactions with key residues more efficiently, leading to drastic remodeling that results in full detoxification of AβOs, while EC only partially detoxifies the AβOs.

## Graphical abstract

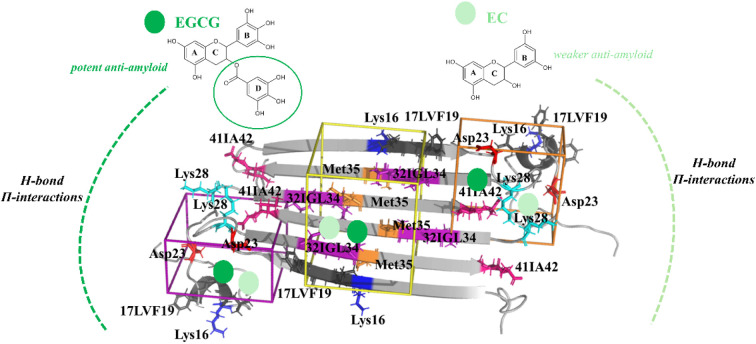

## 1. Introduction

Alzheimer's disease (AD) is the most common neurodegenerative disease (ND) and the leading cause of dementia in the elderly, impacting millions of people worldwide. The primary hallmark of AD is the aggregation and accumulation of the amyloid β (Aβ) peptide, leading to the formation of senile plaques in the brain.^1,2^ However, the implications of Aβ oligomers (AβOs) in cognitive impairment and disease progression are more importantly highlighted than mature fibrils.^3^ The formation of amyloid plaque is a complex chain of nucleation events that produces the most neurotoxic intermediates in the form of soluble oligomers.^4,5^ In detail, the Aβ aggregation pathway is a three-phase process, wherein natively unfolded monomers can self-aggregate into toxic oligomers (nucleation phase), which can then extend into protofibrils (elongation phase) and ultimately mature fibrils (saturation phase).^6^

Soluble AβOs have emerged as key therapeutic targets due to their potent cytotoxic effects and crucial roles in cognitive function in AD.^7–9^ Meanwhile, membrane-embedded AβOs form ion-permeable channels in cellular membranes, leading to disturbances between excitatory and inhibitory neurotransmission.^7^ Numerous findings now suggest that many natural products (NPs) act as inhibitors or modulators of Aβ aggregation pathway. In this context, targeting the most toxic AβOs with NPs may prove to be an effective treatment by preventing their spread.^8,9^ Among the many NPs that have been shown to suppress the toxicity of Aβ aggregates, green tea catechins have been highlighted for their ability to remodel neurotoxic oligomers into non-neurotoxic assemblies.^10^ Furthermore, epigallocatechin-3-gallate (EGCG) possesses abundant evidence of its neuroprotective effects in *in vitro* and *in vivo* AD models.^11,12^

Ahmed *et al.* (2017) demonstrated that EGCG binds at different sites on AβOs surface, inducing a shielding effect by reducing the exposure of hydrophobic residues, which is a key determinant of oligomer cytotoxicity.^13^ The same group also showed that a catechin library could be used to generate an ensemble of AβOs with varying degrees of cytotoxicity *in vitro*.^10^ These studies provided convincing evidence that EGCG remodels AβOs into non-toxic structures, whereas other green tea catechins, such as epicatechin (EC), only partially detoxify oligomers. Furthermore, they showed that EGCG-remodelled oligomers lose their ability to induce neurotoxicity *via* pore formation.

Molecular dynamics (MD) simulation studies have been crucial in unravelling the complex interactions between NPs and amyloid aggregates. These studies have provided valuable insights^14–22^ into the interactions of NPs with protofibrils and fibrils. However, the interactions between NPs and oligomers have not been well studied. The lack of atomic structures of oligomers until recently has contributed to a gap in our understanding.^14^ To date, only a few studies aimed to investigate the binding of NPs into oligomers. These studies typically replicated oligomer structures using dimers, trimers, or tetramers derived from solid amyloid fibril structures. This approach is well limited, as it relies on segments of solid fibrillar structures, which are inherently less toxic than the pore-forming, soluble oligomers they aim to model.^15,16^

Notably, it wasn't until 2020 that Ciudad *et al.* successfully elucidated the first atomic structures of soluble oligomers.^17^ These authors utilized NMR to resolve the structures of AβOs_(1–42),_ revealing them to be tetrameric and composed of two hydrophobic faces of the β-sheet core, with hydrophilic edges. Additionally, these findings of Ciudad *et al.* shed light on the neurotoxic mechanism by which AβOs form lipid-stabilized pores, disrupting neuronal membranes and ion homeostasis.^14^ It is now believed that in neuronal membranes, the hydrophilic edges of the oligomers interact unfavourably with exposed lipid tails, eliciting lipid headgroup reorientation, and leading to pore formation, which alters cellular ion homeostasis.^14,17^

Although the neuroprotective effects of EGCG against AβOs have been well-documented *in vitro*, the specific binding sites and intermolecular interactions underlying EGCG's remodelling of AβOs into non-toxic assemblies remain unclear. In our study, we performed a computational approach combining binding site prediction, molecular docking, and MD simulations to gain insights into the interactions between AβOs and green tea catechins, specifically EGCG and EC. Notably, we utilized the tetrameric structure resolved by Ciudad *et al.* for the first time in this type of study, enabling us to investigate the dynamic interplay between NPs and oligomers at the atomic level.

We found that both catechins bind *via* hydrogen bond (H-bond) and aromatic interactions to three potential binding sites, including two pockets within the hydrophilic edges and a third in the hydrophobic core. Additionally, EGCG formed H-bonds with some key residues for oligomerization, mostly in the hydrophobic oligomer β-sheet core. Since EGCG has been experimentally validated as the catechin with higher anti-amyloid potency, it is likely that the establishment of H-bonds with these key residues play an important role in its remodelling capacity.

## 2. Methods

### 2.1. Binding site prediction

The structure of pore-forming Aβ tetramers obtained from the Protein Data Bank (PDB ID 6RHY) was mapped using the DoGSiteScorer web server (Zentrum für Bioinformatik: Universität Hamburg – Proteins Plus Server), a computational tool for automatic binding site prediction, analysis and druggability assessment.^18^ DoGSiteScorer analyses a protein of interest to detect potential binding pockets and subpockets. Subsequently, it calculates the geometric and physicochemical properties of these pockets and estimates druggability using a support vector machine (SVM). A druggability score between 0 and 1 is then calculated, where a higher score indicates a more druggable pocket. The 3D visualization of mapping analysis of protein binding cavities was carried out using PyMOL version 1.8 (Schrödinger, LLC).

### 2.2. Molecular docking protocol

The structures of EGCG and EGC were constructed using Avogadro software (version 1.2.0),^28^ and their energy-minimized 3D structures were generated using the Merck molecular force field (MMFF94).^19^ Molecular docking was performed using AutoDock Vina (version 1.2.0). The structures of EGCG or EGC were docked to the three promising sites identified as druggable sites by the DoGSiteScorer web server analysis. Three different setups for the grid box were used for molecular docking studies (Table 1). The docking interaction profile was analyzed using Discovery Studio 2021 software (Biovia).

**Table 1:** Grid box parameters used for molecular docking studies

Grid box setup				
“Sites”	Grid box size	Grid box center		
	(*x* × *y* × *z*)	*x*	*y*	*z*
P0	26 × 22 × 24	24.318	−9.09	3.306
P1	24 × 24 × 20	−22.564	0.93	−3.896
P2	24 × 24 × 26	0.386	−4.417	−1.578

### 2.3. Molecular dynamics protocol

MD simulations were performed using the GROMACS 2021.2 package. The ff99SB force field and the TIP3P water model were employed. The protein or docking complex was placed in a truncated octahedron water box using periodic boundary conditions. Overall charge neutrality was preserved by adding 3 K+ ions. Each system was initially energy-minimized using the steepest descent method until convergence was reached, with the maximum force in the system smaller than 1000 kJ mol^−1^ nm^−1^. Subsequently, all systems were subjected to NVT followed by NPT equilibration for 200 ps at 300 K. The cutoff values for the van der Waals and coulombic interactions were set to 1.0 nm. Electrostatic interactions were calculated using the Particle Mesh Ewald (PME) method. We performed two individual 500 ns MD simulations for each equilibrated system at a temperature of 300 K and a pressure of 1 bar under periodic boundary conditions. The global structural stability was assessed using several standard simulation parameters, including the root mean square deviation (RMSD) over backbone atoms, the radius of gyration (*R*_g_), the solvent accessible surface area (SASA), and the root mean square fluctuation (RMSF) over backbone atoms. Our visual analysis of the trajectories was carried out in the Visual Molecular Dynamics (VMD, version 1.9.3) package and the conformations were visualized using Discovery Studio 2021 software (Biovia).

## 3. Results and discussion

### 3.1. Three druggable binding sites are identified within the AβO

The *in vitro* production of amyloid fibrils and other aggregates, formed from chemically synthetic or recombinantly expressed peptides in *Escherichia coli*, has significantly advanced our understanding of amyloid aggregation, revealing the inherent complexity of amyloid aggregates.^20^ These structures often exist in multiple conformations and transient states, which present challenges in obtaining high-resolution structural information.^21–23^ However, in 2020, a major breakthrough was achieved with the elucidation of the first soluble oligomer structure by NMR.^14,17^ Despite this promising discovery reported by Ciudad *et al.*, no studies have yet utilized these elucidated structures to investigate the effects of anti-amyloid compounds on remodelling. To explore this atomic structure, we investigate the binding of EGCG and EC to these oligomers using a computational approach.

**Fig. 1:**
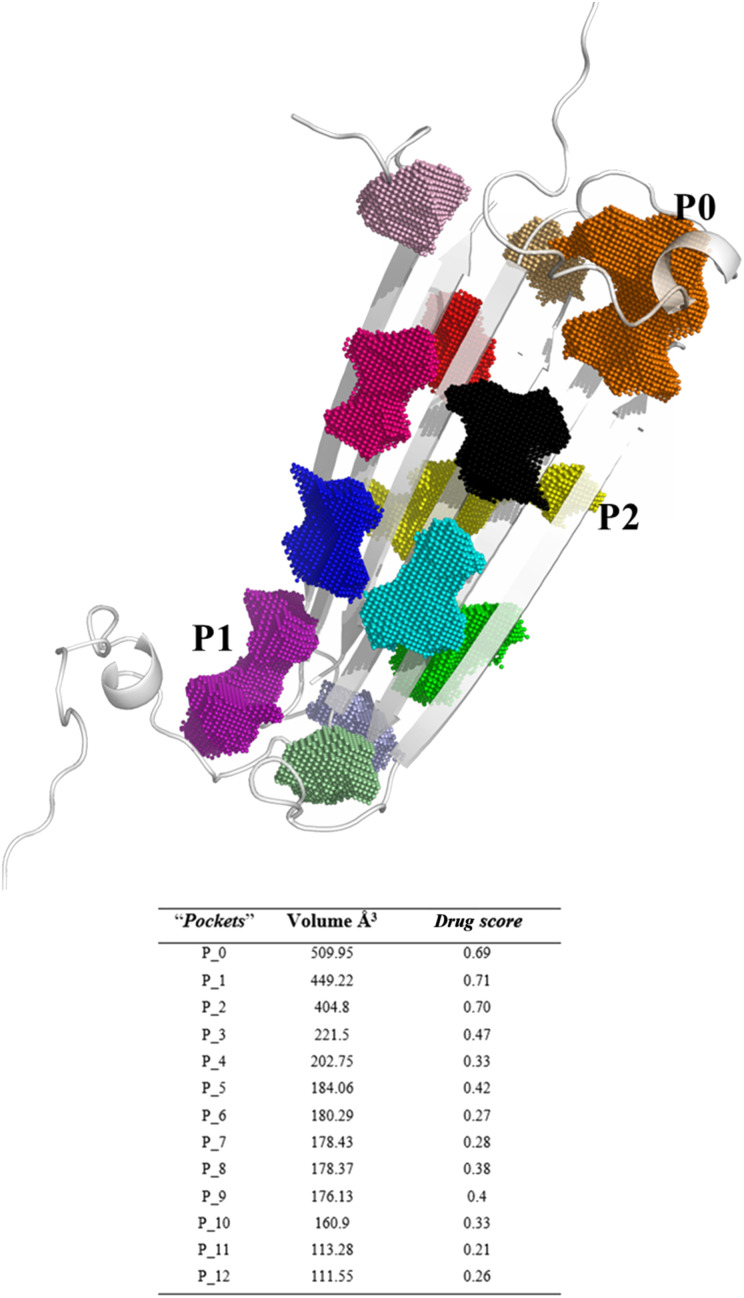
Binding site prediction of AβO using the DoGSiteScorer web server. The pocket mapping analysis identified 13 putative pockets shown as colored dots (P0–P12). The first three pockets estimated to be druggable are highlighted: P0 in orange, P01 in purple, P02 in yellow.

Since the precise binding sites of catechins within the AβO are still unknown, we utilized DoGSiteScorer tool from ProteinsPlus web server in our study. We first investigated the NMR structure of pore forming Aβ tetramers elucidated by Ciudad *et al.* and obtained from the Protein Data Bank (PDB ID 6RHY) for ligand binding site prediction.^18^ The pocket mapping analysis revealed 13 putative pockets (named from P0 to P12 by the server) on the surface of this AβO structure (Fig. 1). Ten pockets (P04 to P013) displayed low druggability estimation with a drug score ≤ 0.6.

**Fig. 2:**
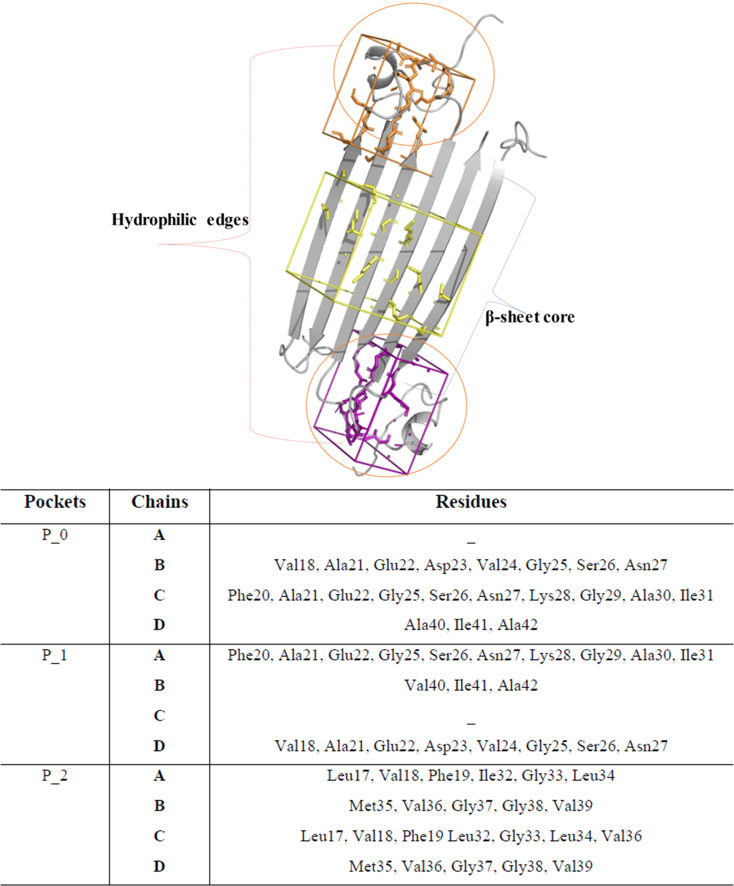
Druggable binding sites identified in AβOs. P0 (orange) and P1 (purple) are found in the hydrophilic edges, while P2 (yellow) is in the hydrophobic β-sheet core.

Notably, P0 and P1, pockets with the highest drug score, were found in the hydrophilic edges of the oligomers, which are crucial for establishing interactions with cell membranes, while P2 is part of the hydrophobic β-sheet core (Fig. 2). As mentioned before, Ciudad *et al.* have proposed a central role for the hydrophilic edges in membrane disruptions.^17^ Based on our *in silico* binding prediction study, which identified druggable pockets in the hydrophilic edges crucial for neurotoxicity of AβOs through induced pore formation, as proposed by Ciudad *et al.*,^17^ we speculate that EGCG's ability to alleviate AβOs neurotoxicity by preventing membrane disruption,^21^ as observed *in vitro* by Ahmed *et al.*,^10^ could be attributed to its targeting of the hydrophilic edges. Binding at this site may help maintain membrane integrity by preventing interactions between oligomers and the exposed lipid tails of the membrane.

Concerning P2, it is a hydrophobic pocket in the β-sheet core. Importantly, the molecular mechanism for the EGCG-induced toxic to nontoxic remodelling of AβOs has been directly related to the regulation of solvent exposure to hydrophobic surfaces.^13^ Recently, Im *et al.* (2023)^24^ identified key domains involved in Aβ oligomerization within the oligomer β-sheet core using designed point mutants of Aβ_42_. These key domains including 17LVF19 and 32IGL34, which are found in P2, now represent promising target domains for the design of novel therapeutic agents.^24^ Taken together, these findings suggest that targeting the P2 in the β-sheet core could be a feasible approach to perturbing fibrillar aggregation.

Ultimately, we believe that future *in vitro* studies assessing pore formation induced by AβOs, using designed point mutants of Aβ42 targeting key residues of hydrophilic edges, will help validate their role in the neurotoxicity mechanism of AβOs. Also, performing the same approach in the presence of green tea catechins, particularly, EGCG, will elucidate whether the mechanisms by which EGCG remodelling AβOs are dependent on interactions with some key residues found in the hydrophilic edges.

### 3.2. Docking of green tea catechins to the three binding sites

**Fig. 3:**
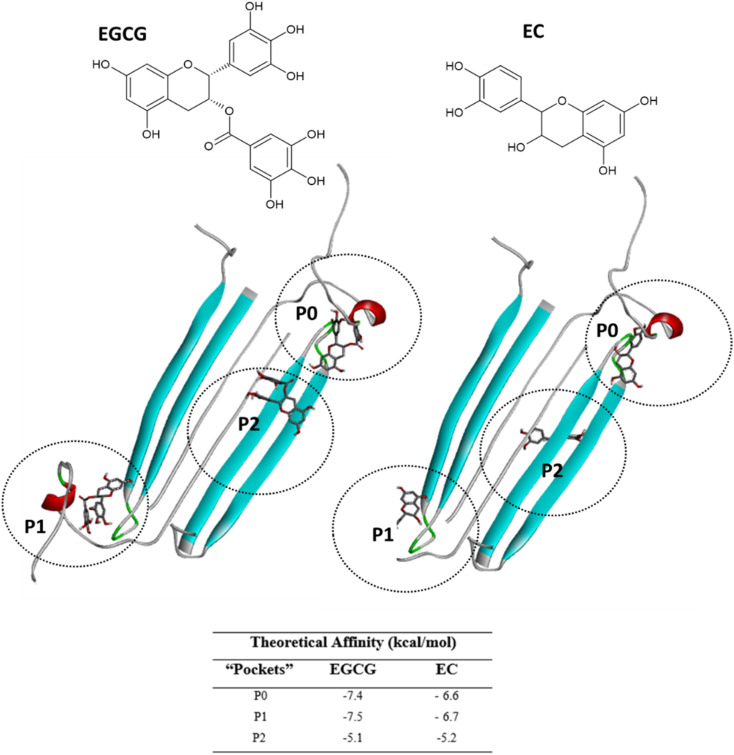
EGCG (left) or EC (right) bound to three main binding sites identified on the AβO surface. Ligands are represented in gray sticks and AβO is shown as cyan cartoon (with negatively charged residues in red and polar residues in green) (with negatively charged residues in red and polar residues in green).

Next, we selected (P0, P01, and P02) as promising binding sites for further investigation. Then, we used Autodock vina software to dock the structures of EGCG and EC into these three pockets. Our docking simulations suggested that EGCG and EC have similar binding energies for P0, P1 and P2 pockets, but both ligands were docked with lower affinity within pocket P2 into the hydrophobic core than within the hydrophilic edges (Fig. 3). Nevertheless, *in vitro* studies by Sironi *et al.* (2014) have suggested that EGCG has higher affinity for AβOs than others catechins lacking the gallate moiety.^25^ These authors found that while the gallate moiety is not essential for the binding of catechins to AβOs, it seems to increase the affinity for them.

**Fig. 4:**
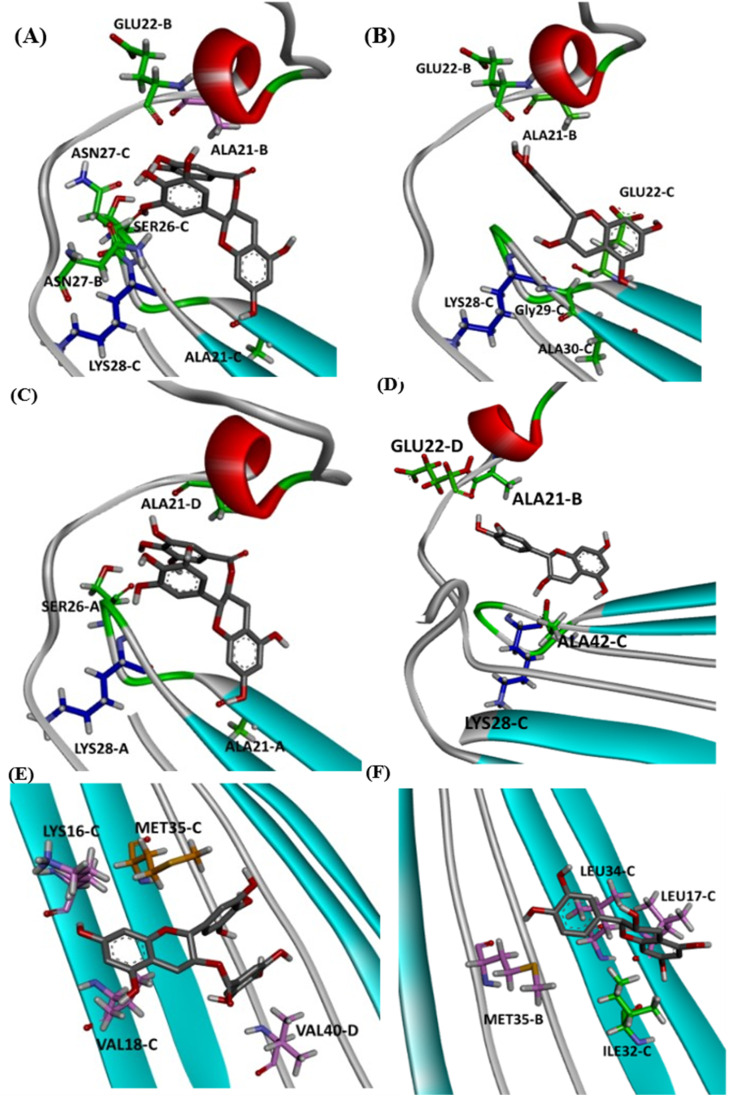
Intermolecular interactions observed in the complexes obtained between AβO and EGCG (left) or EC (right) docked in P0 (A and B), P1 (C and D), and P2 (E and F). The oligomer structure is shown as cartoon, and ligands are represented as gray sticks. Positive charged residues are represented as blue sticks, while negatively charged residues are shown as red sticks. Residues forming H-bonds are illustrated as green sticks, and aromatic interactions are represented by orange sticks (π-sulfur) and light-pink sticks (π-alkyl).

Detailed docking pose interaction analysis suggests that in the P0, EGCG formed H-bonds with Ser26 and Asn27 residues through its vicinal trihydroxy groups in the B ring and gallate group, whereas EC did not interact with these residues into the P0 in the hydrophilic edge (Fig. 4a and b). In the other hydrophilic edge (P1), similar interactions were observed (Fig. 4c and d). Additionally, the residues Asp23 and Lys28, found in the hydrophilic edges, form a salt-bridge, which is required for the conformational change from α-helix to β-sheet during the aggregation process.^26,27^ Then, the EGCG presence into P0 or P1 could disturb the aggregation formation by avoiding the Asp23–Lys28 salt-bridge establishment.

In the P2, EGCG and EC did not dock into the same face of the hydrophobic β-sheet core. EGCG engaged in aromatic interactions with the residues of chain C: Lys16-C and Met35-C, which are key residues in the Aβ aggregation. In contrast, the less effective catechin, EC interacted with Met35-B (Fig. 4e and f). The single Met along the Aβ sequence, Met35, is a key residue for oligomer formation, and the oxidation of this residue in the Aβ monomer is an anti-amyloid strategy to delay aggregation.^28,29^ Additionally, Lys residues have been reported to play a key role in the Aβ aggregation. *In vitro* studies have shown that the substitution of Lys at position 16 by Ala suppresses the amyloid toxicity.^30^ We also observed the establishment of intermolecular interactions with some residues from the critical domains 17LVF19 and 32IGL34, which facilitate Aβ oligomerization.^24^ EGCG formed a π-alkyl interaction with Val18-C, while EC interacted with Leu17-C *via* π-alkyl, in addition to establishing a H-bond with Ile32-C.

Notably, catechins serve as useful tools in reducing the neurotoxicity of oligomers by modulating their interactions with membranes.^31^ Ahmed *et al.* (2019)^10^ found that AβOs formed in the presence of catechins are significantly less toxic. However, EGCG exhibits a higher remodelling capacity than EC; thus, EGCG-remodelled oligomers are less neurotoxic than EC-remodelled oligomers.^10^ Taken together, our docking results and these previous experimental findings indicate that EGCG and EC bind to AβOs at multiple sites. In addition to aromatic interactions with residues in the hydrophobic core, which are crucial for aggregation and neurotoxicity, EGCG demonstrates higher affinity binding at the hydrophilic edges of AβOs. This binding may prevent the formation of the interactions between oligomer and cellular membranes.

### 3.3. Catechins remain stable docked into the potential binding sites

MD simulations have been broadly applied to provide insights into the detailed interactions between green tea catechins and monomers/protofibrils/fibrils of amyloidogenic proteins.^32–37^ Zhan *et al.* investigated by MD simulations how EGCG and epicatechin gallate (EGC) interact with full-length Aβ42 protofibril, disrupting this structure.^34^ The results showed that EGCG displays a higher disruptive capacity than EGC *via* H-bond and aromatic interactions including π–π stacking and cation–π interactions. The authors thus suggested that the gallic acid ester group of EGCG plays a crucial role in disruptive mechanisms since EGCG contains an extra gallic acid ester group compared to EGC.^34^ Moreover, a combination of extended MD simulation, *ab initio* calculations, and *in vitro* immuno-infrared analyses revealed that EGCG disrupts the interchain hydrogen bonds and salt bridges, which are crucial for the fibril structure and shape of Aβ. The same study found that the interactions of EGCG are dominated by only a few residues in the fibrils, including hydrophobic π–π interactions with aromatic rings of side chains and hydrophilic interactions with the backbone of Aβ.^38^

All studies mentioned above have used full or edited 3D structures of solid fibrillar species of Aβ with classical U-shaped or S-shaped conformations, in which two interacting protofilaments typically form an amyloid arrangement. These structures are markedly distinct from the two hydrophobic faces of the planar structure of tetrameric soluble AβOs investigated in our simulations.^17^

Oligomers are small, soluble, and freely diffusible protein assemblies that do not adopt the fibril structure but instead have a more globular shape.^20^ They are often categorized into “on-pathway” and “off-pathway” oligomers, based on their ability to further grow into mature fibrils.^39^ Additionally, oligomers can also be released by mature fibrils after their formation.^40^ Among Aβ species, structured oligomers are proposed to be more toxic than fibrils because they interact with cell membranes, causing pore formation, which results in the leakage of ions, disruption of cellular calcium balance, and loss of membrane potential.^41^

The amyloid pore hypothesis was first proposed in 1993 by Arispe and co-workers.^42^ The authors suggested that Aβ promotes neurotoxicity by forming pores in neuronal membranes with channel-like activity. Aβ aggregation pathway is the core biological hallmark of AD and is considered a promising target for the development of disease-modifying therapies.^43^ However, recent late-stage trials with AD patients have revealed that only agents that target soluble oligomeric Aβ show clinical efficacy in AD.^44^

**Fig. 5:**
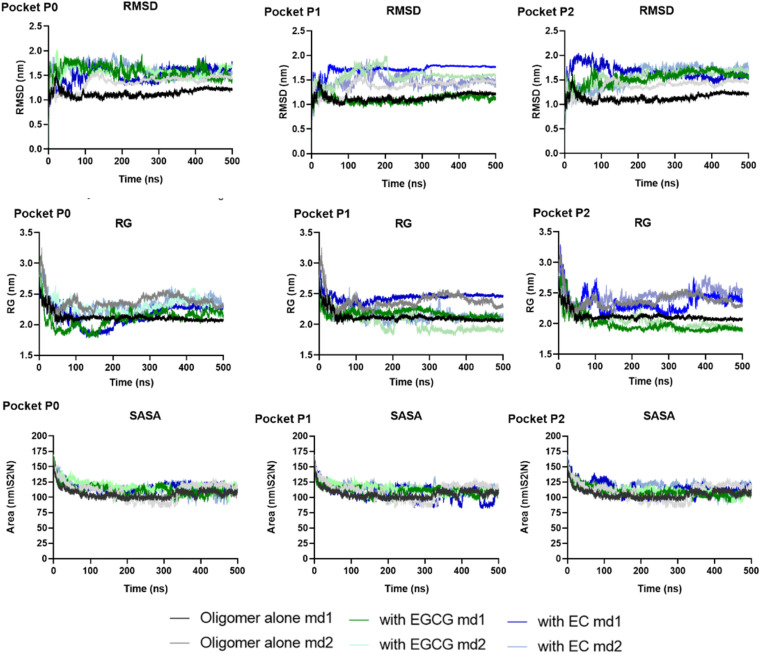
The global structural stability of two independent replicas of systems examined through RMSD over backbone atoms, *R*_g_, and SASA.

Herein, we performed MD simulations of each EGCG-AβO and EC-AβO complex using GROMACS 20.2 package to investigate the stability of catechins binding to the three binding sites identified earlier. Firstly, we assessed the global structural stability of all systems by calculating several parameters, including RMSD over backbone atoms, *R*_g_, and SASA (Fig. 5 and Table S1†). Following equilibration, we did not observe any major changes caused by the presence of catechins in the binding sites for the systems with ligands. Additionally, the standard simulation parameters indicated reasonable stability during the 500 ns simulations for all examined systems. The average values of RMSD over backbone atoms ranged between 1 and 2 nm, with a mean value for *R*_g_ ranging from 2.00 to 2.45 nm.

The backbone RMSF values per residue of each chain were calculated to assess protein chain flexibility (Fig. S1 and S2†). The highest fluctuations were observed for chain D for all systems, whether in the presence or absence of ligands. Additionally, chains A, B, and C exhibited higher fluctuations in the presence of any ligand. Specifically, the RMSF for A–C chains ranged from 0.3 to 0.5 nm in the absence of ligands, whereas in the presence of EGCG or EC the RMSF values ranged to 0.3–0.9 (Tables S2 and S3†). This suggests that the presence of catechins in the pockets identified likely perturbed the arrangement of individual β-sheets, increasing flexibility.

**Fig. 6:**
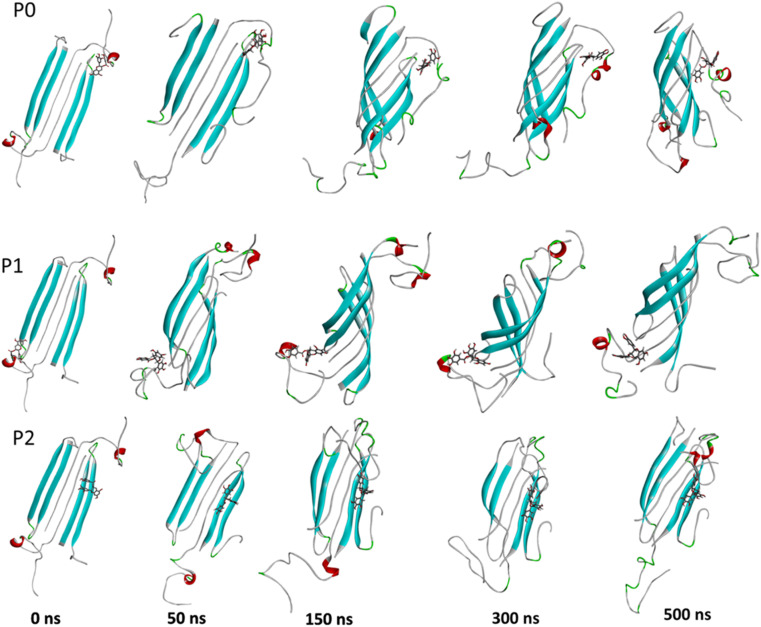
Snapshots of the Aβ oligomer in the presence of EGCG docked in P0, P1, and P2 at 0, 50, 150, 300, and 500 ns for md1. The oligomer structure is represented in cyan (with negatively charged residues in red and polar residues in green), and EGCG is represented as gray sticks.

**Fig. 7:**
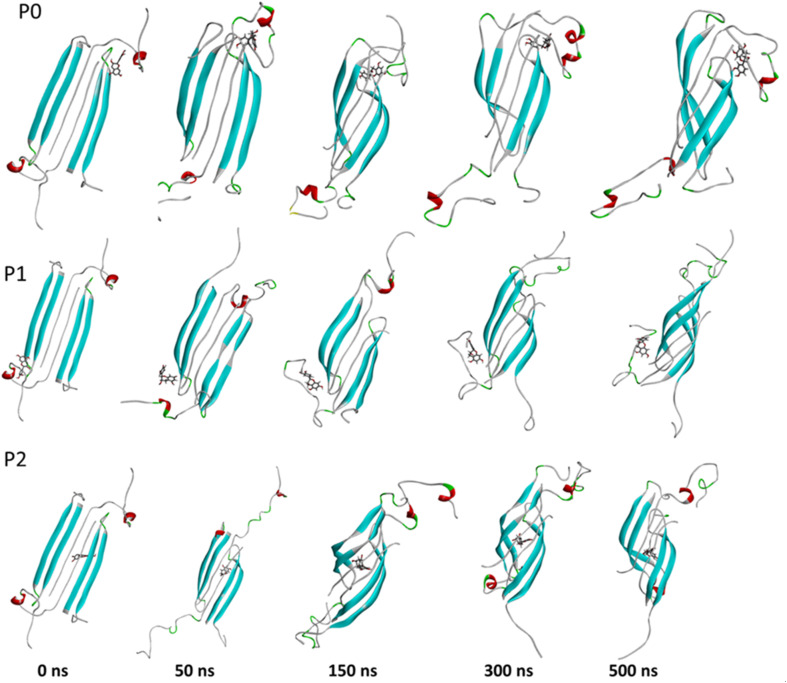
Snapshots of the Aβ oligomer in the presence of EC docked in P0, P1, and P2 at 0, 50, 150, 300, and 500 ns for md1. The oligomer structure is represented in cyan (with negatively charged residues in red and polar residues in green), and EC is represented as gray sticks.

The complexes' stability was also confirmed by visual examination. Snapshots of md1 at 50, 150, 300 and 500 ns were analysed. Both catechins remained stably docked into each binding site throughout 500 ns simulation (Fig. 6 and 7). We speculate that the binding of EGCG in these three binding sites is part of its remodelling mechanism. In addition, Ahmed *et al.* (2019)^10^ demonstrated experimentally that the binding of EGCG or EC remodels the oligomer, but the structural changes were more prominent in EGCG-remodelled AβOs.^10^ Thus, it seems plausible that the presence of catechins, mostly EGCG, can disturb interchain interactions, remodelling the β-sheet oligomer interface.

### 3.4. EGCG forms H-bond with key residues more efficiently

**Fig. 8:**
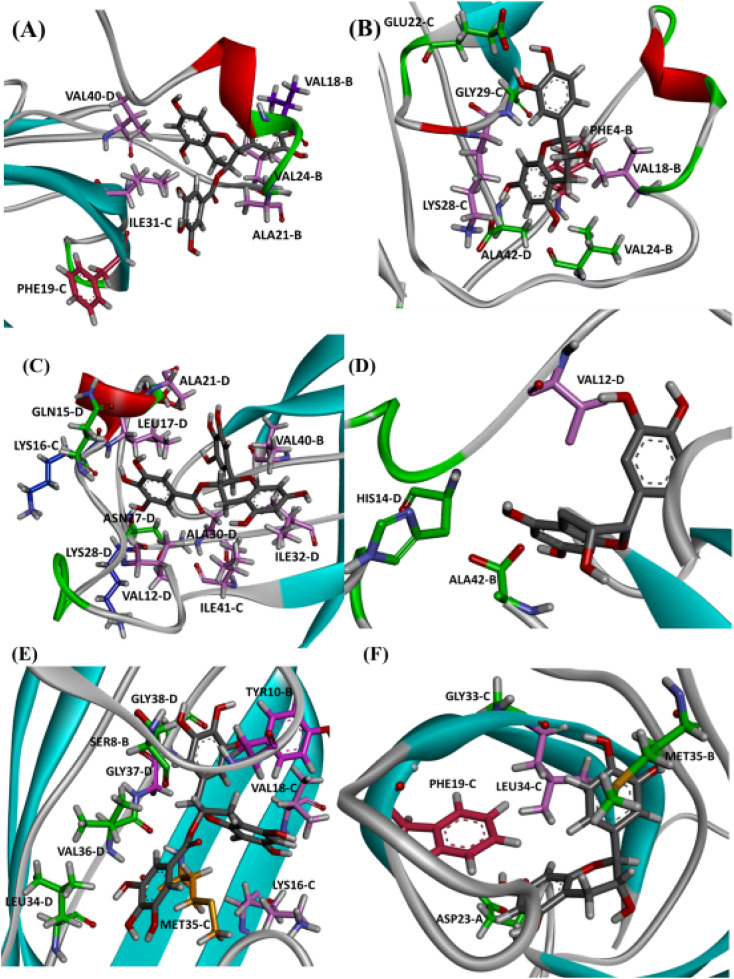
Intermolecular interactions observed in the final state of complex EGCG-AβO and EC-AβO docked in site P0 (A), P1 (B), and P2 (C) for md1. The oligomer structure is shown in cyan, and the ligands are represented as gray sticks. Residues involved in H-bonding are shown as green sticks, while residues involved in electrostatic interactions are represented as light pink (π-alkyl), hot pink (π-stacking), and orange (π-sulfur).

It is well known that anti-amyloid NPs interfere with electrostatic and hydrophobic interactions that stabilize β-sheets in the aggregates, due to the establishment of intermolecular interactions with sidechain or backbone residues of the protein such as H-bond, π–π interactions, or ionic interactions.^45^ To gain insight into the interaction forces between oxidized EGCG or EC and AβOs, we calculated the distance between EGCG or EC and the residues in the potential binding sites (Tables S4 and S5†). Throughout the MD simulations, both catechins remained within 0.35 Å from some key residues, including residues from the critical domains 17LVF19, 32IGL34, and 41IA42, involved in oligomerization.^24^ The binding of EGCG or EC into these domains likely contributes to the remodelling effect, disturbing interchain interactions that are important to the aberrant aggregation process. Additionally, detailed molecular interaction analysis in the final state of each complex, EGCG-AβO and EC-AβO, for md1 reveals the establishment of intermolecular interactions such as H-bonds and π interactions between catechins and key residues, including Leu17, Val18, Phe19, Asp23, Lys28, Leu34, Met35, and Ala42 (Fig. 8). We also investigated the possible formation of H-bonds by calculating the H-bond number between catechins and the AβO (Fig. S3†).

**Fig. 9:**
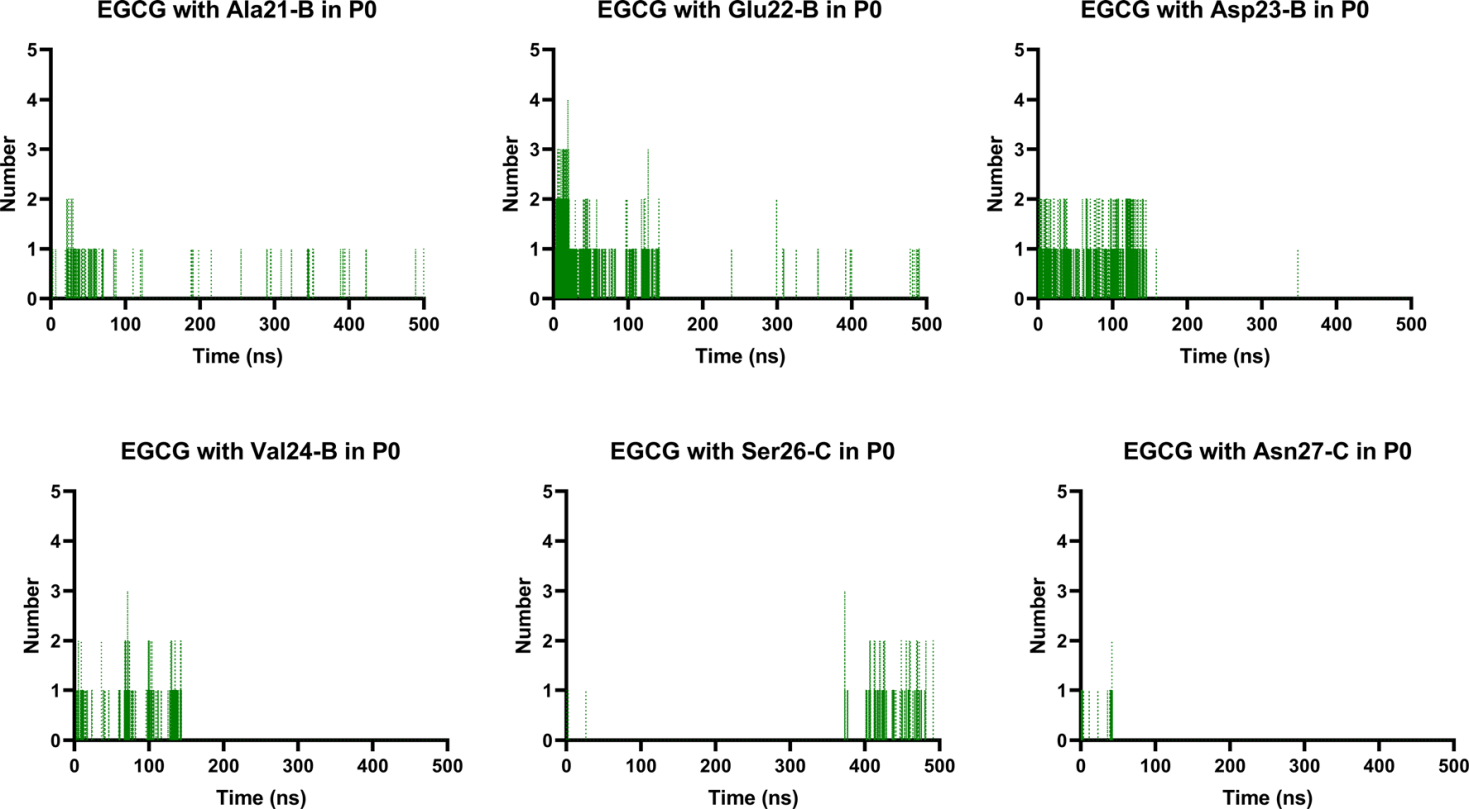
Prevalent H-bonds in P0 for the EGCG-remodelled oligomer calculated over 500 ns for md1.

Finally, we analysed the entire trajectories of each system to calculate the occupancy rates for H-bonds with key residues over the 500 000 coordinate frames (Table S6†). In P0, we found that catechins preferentially established H-bonds with polar and negatively charged residues from chains B and C, including Glu22, Asp23, Ser26, and Asn27. However, more prevalent residues were identified as involved in H-bonding in the EGCG-remodelled oligomer; also, only EGCG formed H-bonds with Asp23 over the 500 ns simulation (Fig. 9 and S4†).

**Fig. 10:**
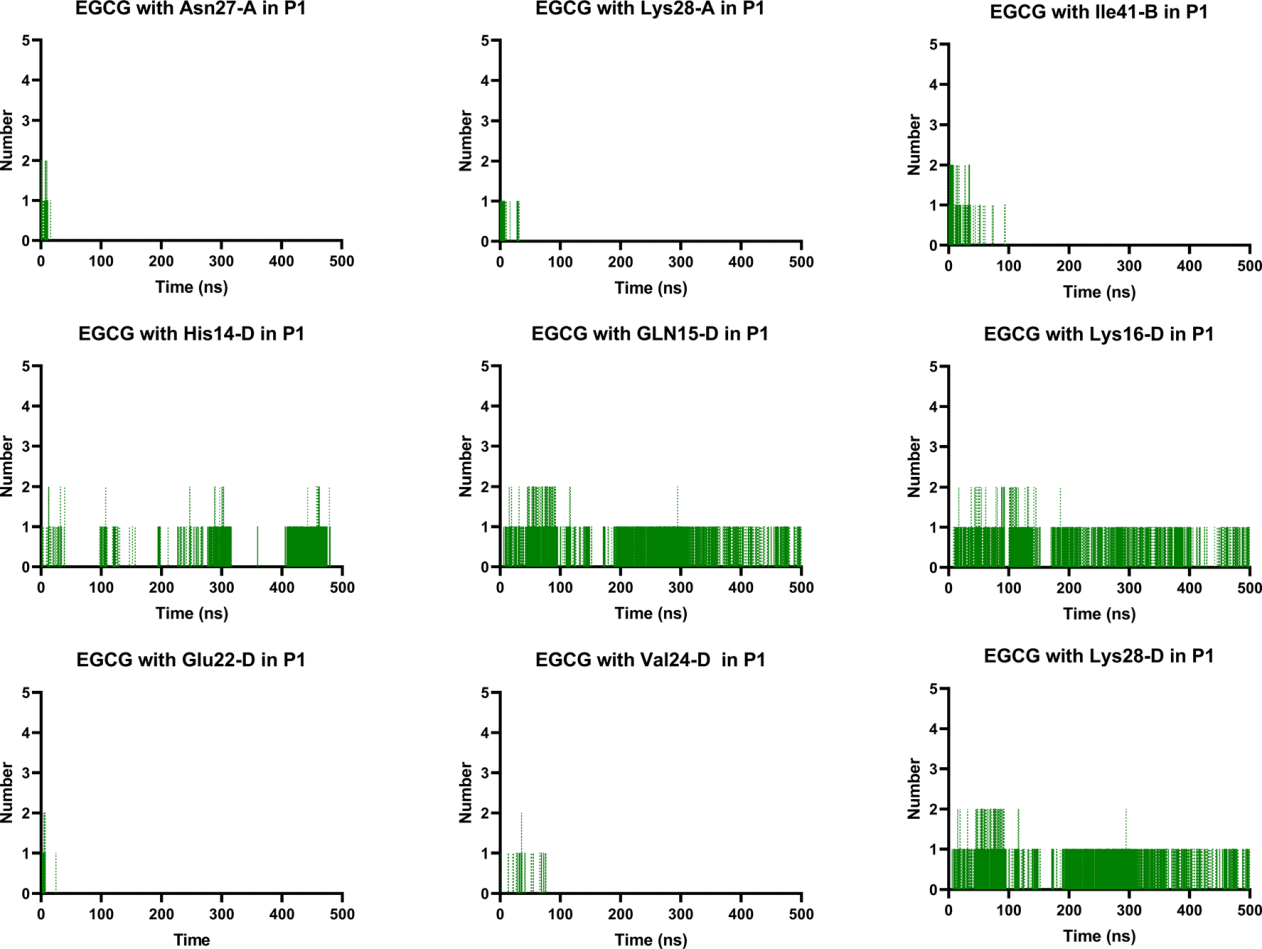
Prevalent H-bonds in P1 for EGCG-remodelled oligomer calculated over 500 ns for md1.

Notably, the formation of a salt bridge involving the side chains of residues Asp23 and Lys28 is a stabilizing feature of Aβ aggregates.^27^ In P1, we found that EGCG established a H-bond with Lys28-D. Thus, it seems plausible that the formation of interactions between EGCG and Asp23 or Lys28 may play an important role in disturbing this salt bridge and consequently could destabilize the AβO. Additionally, an H-bond between EGCG and another Lys residue in Aβ, Lys16, was also noted whereas, in the EC-remodelled oligomer no H-bond with Lys residues was identified (Fig. 10 and S5†). Lys residues are believed to have a key role in the self-assembly of Aβ, participating in a combination of hydrophobic and electrostatic interactions. Hence, Lys16 and Lys28 are considered promising residues as targets for anti-amyloid therapeutic agents.^30^

**Fig. 11:**
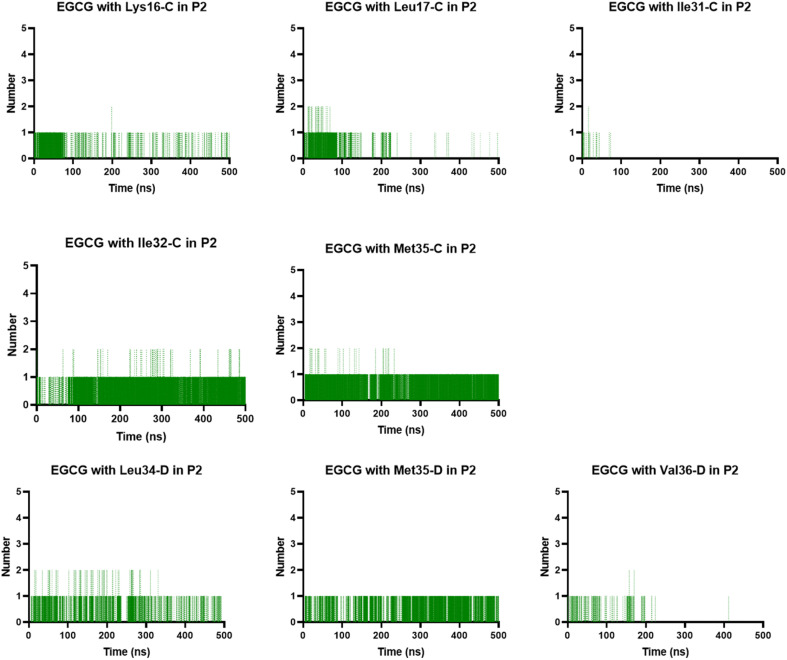
Prevalent H-bonds in P2 for the EGCG-remodelled oligomer calculated over 500 ns for md1.

In the P2, we also found H-bonds between EGCG and Lys16 (Fig. 11 and S6†). Importantly, this Lys 16 residue is found adjacent to the central hydrophobic cluster (residues 17–21), which is a key region in Aβ fibrillogenesis.^46^ Our H-bond analysis also revealed that only in the EGCG-remodelled AβOs did the establishment of H-bond occur across the simulation with the following key residues of β-sheet core: Ile32, Leu34, and Met35 (from chains C and D). Herein, we speculate that EGCG may be a more potent anti-amyloid compound than EC due to its ability to form H-bonds with these key residues more efficiently. Likewise, the formation of H-bonds plays a key role in the mechanism by which EGCG remodels toxic oligomers into non-toxic ones.

## 4. Conclusion

The first atomic structures of AβOs, elucidated by Ciudad *et al.*, represent a significant advancement that has shed light on the neurotoxic mechanisms of AβOs. However, this structure has been relatively unexplored in investigations into the mechanisms underlying the action of anti-amyloid compounds. In this study, we investigated the remodelling of AβOs into non-toxic species by the potent anti-amyloid EGCG, utilizing this soluble oligomer structure. Previous studies have primarily relied on structures extracted from solid fibril conformations to mimic oligomer structures.

In summary, our study benefits from the utilization of this experimentally elucidated real structure of the most toxic amyloid form for computational investigations of anti-amyloid remodelling. We explored the binding of EGCG to AβOs to uncover the intermolecular interactions involved in AβO remodelling by EGCG. Our docking studies, coupled with MD simulations, unveiled that EGCG binds more efficiently to two pockets located on the hydrophilic edges, compared to the less effective anti-amyloid EC. These hydrophilic edges are proposed as key structures for incorporation of the AβOs into the cellular membranes.^17^ Moreover, the presence of EGCG in the hydrophilic edges may prevent pore formation by inhibiting oligomer interaction with cell membranes, consistent with experimental observations.^10^

In the third pocket found in the β-sheet core of AβO, there are some key residues, such as Lys16, Leu17, Val18, Ile32, Leu34, and Met35, all of which are important for fibril formation.^24,30,47^ Ultimately, we show that EGCG forms H-bonds with these key residues more efficiently than EC. These interactions may disrupt fibrillation, redirecting the Aβ aggregation pathway towards off-pathway aggregates. Our findings suggest that the presence of the gallate moiety as well as three vicinal hydroxyl groups in EGCG's aromatic rings increases the formation of H-bond with key residues and it is likely critical in remodelling.

The unique arrangement of side chains within amyloid aggregates forms the core of the β-sheet structure.^20^ In EGCG-remodelled AβOs, the interaction of EGCG with the oligomer's β-sheet core can disrupt the aromatic hydrophobic core, thereby interfering with crucial interchain interactions necessary for amyloid formation, order, and stability. This disruption of interchain aromatic interactions likely underlies the mechanism of action of EGCG in remodelling AβOs.

In conclusion, our computational approach provides insights into the remodelling of AβOs by EGCG and identifies potential binding sites in the AβOs. Further experimental validation is essential to verify their functional significance as promising hot spots for developing therapeutic candidates targeting oligomer neurotoxicity. Our findings highlight the potential of these NMR pore-forming oligomer structures for future exploration in molecular modelling, medicinal chemistry, and drug discovery studies.

## Data availability

The data supporting this article have been included as part of the ESI.†

## Author contributions

P. B. G. conceptualized and designed the study, conducted the *in silico* investigations, analysed the data, and drafted the manuscript. Y. C. and A. C. R. S. supervised the study and contributed to manuscript revisions. All authors have read and agreed to the published version of the manuscript.

## Conflicts of interest

The authors declare no competing financial interest.

## Supplementary Material

RA-014-D4RA03343D-s001

RA-014-D4RA03343D-s002
