# Supplementary material for: Understanding the mechanisms of green tea EGCG against amyloid β oligomer neurotoxicity through computational studies
Source: RSC Adv. 2024 Jul 16;14(31):22525–39. doi: 10.1039/d4ra03343d (PMC11251396; doi:10.1039/d4ra03343d)
Supplement: RA-014-D4RA03343D-s001 [file RA-014-D4RA03343D-s001.pdf]

Electronic Supplementary Material (ESI) for RSC Advances.
This journal is © The Royal Society of Chemistry 2024

DATA AVAILABILITY

Understanding the Mechanisms of Green Tea EGCG against Amyloid β Oligomer Neurotoxicity through Computational Studies

Priscila Baltazar Gonçalves, Yraima Cordeiro and Ana Carolina Rennó Sodero

Data Availability Statement:

Data for this article, including all computational simulations performed, are available at the Zenodo Repository at the following links:

Molecular Docking Data available in:

Docking Studies of Green Tea Catechins with Amyloid β Peptide Oligomers (zenodo.org)

Molecular Dynamics Data available in:

Oligomer alone (md1) (zenodo.org)

Oligomer alone (md2) (zenodo.org)

EGCG-oligomer in P0 (md1) (zenodo.org)

EGCG-oligomer in P0 (md2) (zenodo.org)

EGCG-oligomer in P1 (md1) (zenodo.org)

EGCG-oligomer in P1 (md2) (zenodo.org)

EGCG-oligomer in P2 (md1) (zenodo.org)

EGCG-oligomer in P2 (md2) (zenodo.org)

EC-oligomer in P0 (md1) (zenodo.org)

EC-oligomer in P0 (md2) (zenodo.org)

EC-oligomer in P1 (md1) (zenodo.org)

EC-oligomer in P1 (md2) (zenodo.org)

EC-oligomer in P2 (md1) (zenodo.org)

EC-oligomer in P2 (md2) (zenodo.org)
