# Supplementary material for: Understanding the mechanisms of green tea EGCG against amyloid β oligomer neurotoxicity through computational studies
Source: RSC Adv. 2024 Jul 16;14(31):22525–39. doi: 10.1039/d4ra03343d (PMC11251396; doi:10.1039/d4ra03343d)
Supplement: RA-014-D4RA03343D-s002 [file RA-014-D4RA03343D-s002.pdf]

Electronic Supplementary Material (ESI) for RSC Advances.
This journal is © The Royal Society of Chemistry 2024

SUPPORTING INFORMATION

Understanding the Mechanisms of Green Tea EGCG against Amyloid β Oligomer Neurotoxicity through Computational Studies

Priscila Baltazar Gonçalves, Yraima Cordeiro and Ana Carolina Rennó Sodero

Table S1. Average values of backbone RMSD, SASA, and RG from MD trajectories for two replicas of each system.

Parameters	***Oligomer***		***Sites***	***with EGCG***		***with EC***	
	md1	md2		md1	md2	md1	md2
**RMSD (nm)**	1.14	1.36	P0	1.58	1.56	1.51	1.60
			P1	1.10	1.58	1.71	1.47
			P2	1.54	1.54	1.65	1.56
**SASA (nm\S2\N)**	105.59	110.11	P0	111.47	118.60	115.31	116.17
			P1	110.21	114.95	107.43	116.28
			P2	109.58	110.61	114.36	116.28
**RG (nm)**	2.12	2.38	P0	2.10	2.32	2.15	2.30
			P1	2.18	2.32	2.44	2.15
			P2	2.00	2.02	2.35	2.45

Table S2. Average values of backbone RMSF (nm) per residues for each oligomer chain in two independent replicas of systems without ligands.

Chains	***without ligand***	
	md1	md2
A	0.30	0.38
B	0.42	0.51
C	0.30	0.38
D	0.24	0.68

Table S3. Average values of backbone RMSF (nm) per residues for each oligomer chain for two independent replicas of systems with ligands.

Pockets	***Chains***	***with EGCG***		***with EC***	
P0		md1	md2	md1	md2
	A	0.49	0.72	0.41	0.58
	B	0.47	0.64	0.45	0.63
	C	0.45	0.54	0.41	0.53
	D	0.85	0.86	0.80	0.72
P1	A	0.31	0.60	0.38	0.54
	B	0.37	0.69	0.50	0.73
	C	0.32	0.67	0.41	0.45
	D	0.37	1.02	0.51	0.47
P2	A	0.30	0.56	0.62	0.94
	B	0.35	0.68	0.81	0.99
	C	0.47	0.56	0.84	0.83
	D	0.71	0.56	1.05	0.91

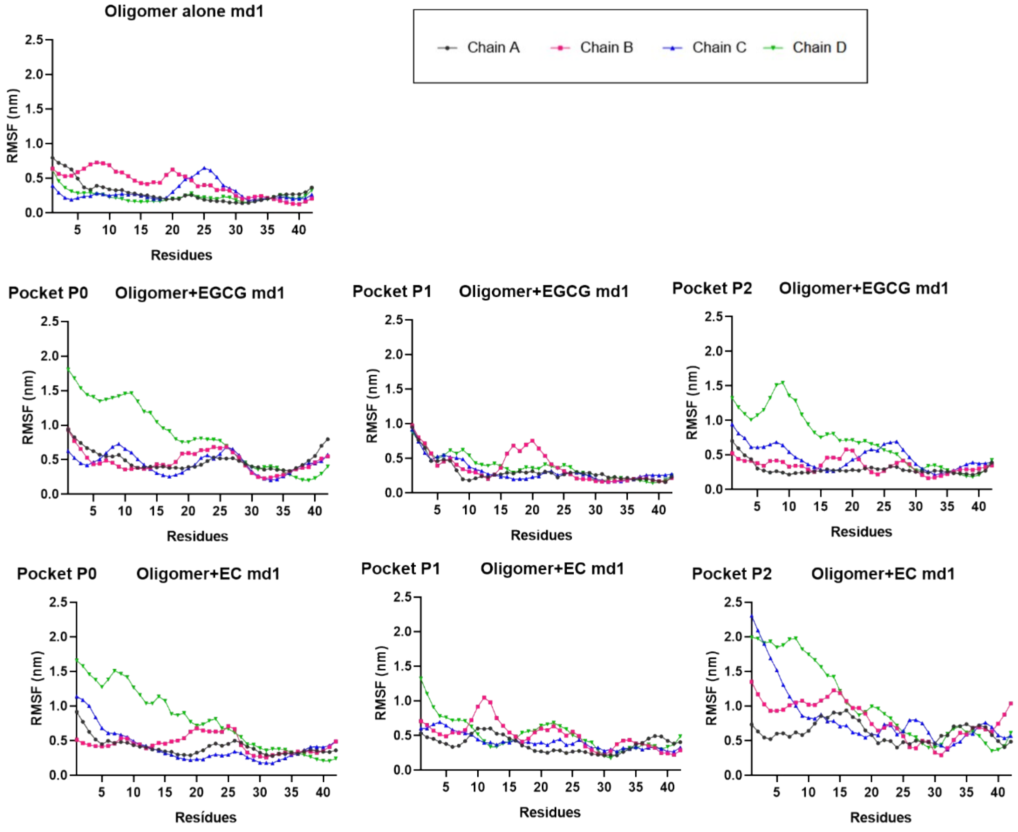

Figure S1. The backbone RMSF calculation per amino acid residues for each oligomer chain for md1.

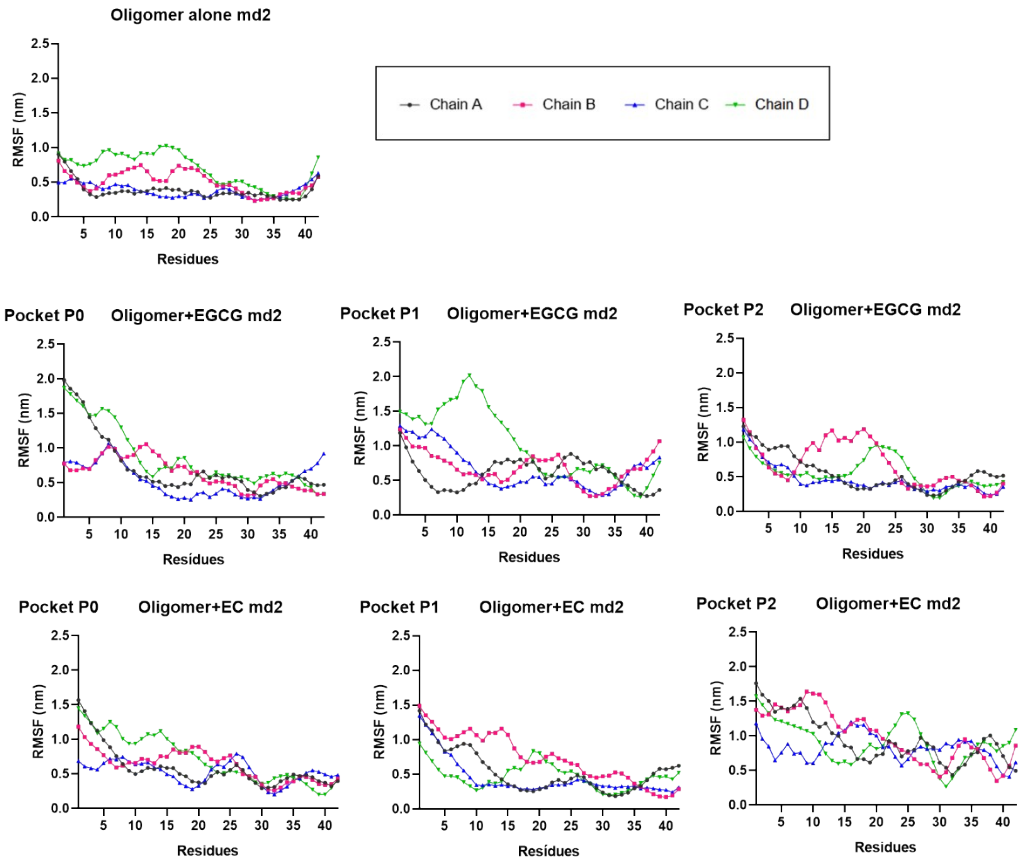

Figure S2. The backbone RMSF calculation per amino acid residues for each oligomer chain for md2.

Table S4. *Distances between EGCG and specific hotspots residues over 500 ns simulation*.

Pockets	Residues	Chains	Distance (Å)*		Distance within 0.35 Å**	
			Md1	Md2	Md1	Md2
P0	Val18	B	1.71	2.85	75.39%	88.31%
	Ala21	B	4.21	4.46	23.10%	16.36%
	Glu22	B	4.41	3.84	28.97%	48.33%
	Asp23	B	5.80	2.45	23.50%	98.71%
	Val24	B	6.26	2.67	17.91%	90.96%
	Gly25	B	7.90	7.0	0.99%	0.002%
	Ser26	B	7.77	7.36	0.28%	0.008%
	Asn27	B	8.12	6.62	0.13%	4.06%
	Phe20	C	2.59	4.80	95.44%	12.91%
	Ala21	C	6.43	8.19	7.79%	3.26%
	Glu22	C	7.81	9.24	2.51%	1.40%
	Gly25	C	10.1	2.99	2.54%	88.28%
	Ser26	C	8.24	2.73	12.13%	95.47%
	Asn27	C	4.61	2.47	36.85%	98.79%
	Lys28	C	2.77	2.87	91.47%	93.47%
	Gly29	C	3.42	3.94	66.59%	65.71%
	Ala30	C	2.62	6.05	98.95%	0.10%
	Ile31	C	2.71	6.83	93.36%	0.03%
	Val40	D	2.51	4.09	97.59%	70.27%
	Ile41	D	0.97	9.73	100%	0%
	Ala42	D	1.09	0	100%	100%
P1	Phe20	A	13.4	11.8	0.008%	12.35%
	Ala21	A	13.0	10.4	0.73%	9.28%
	Glu22	A	15.4	11.0	1.73%	35.03%
	Gly25	A	10.5	8.04	0.11%	28.94%
	Ser26	A	10.2	8.48	1.23%	38.59%
	Asn27	A	11.8	10.3	2.42%	31.78%
	Lys28	A	11.6	11.1	3.65%	30.45%
	Gly29	A	11.8	10.6	5.85%	25.73%
	Ala30	A	9.95	11.1	5.1%	0.002%
	Ile31	A	9.17	12.6	6.27%	0
	Val40	B	2.56	12.1	94.23%	0
	Ile41	B	4.75	9.15	22.25%	0.11%
	Ala42	B	4.99	9.48	15.36%	0.06%
	Val18	D	5.52	3.90	6.1%	58.09%
	Ala21	D	10.2	6.96	0.04%	8.57%
	Glu22	D	9.16	4.71	1.72%	46.99%
	Asp23	D	7.52	4.86	0.04%	59.93%
	Val24	D	10.3	4.78	0.35%	30.54%
	Gly25	D	10.1	7.16	0.09%	6.10%
	Ser26	D	7.28	8.48	1.7%	38.60%
	Asn27	D	9.87	10.4	0.75%	0.02%
P2	Leu17	A	3.61	12	46.74%	0
	Val18	A	13.9	14.9	0	0
	Phe19	A	10.6	11.4	0	0
	Ile32	A	8.35	10.8	0	0

Gly33	A	10.3	12.6	0	0
Leu34	A	8.94	9.38	0	0
Met35	B	5.38	5.39	0.21%	0
Val36	B	6.13	8.19	0.31%	0.002%
Met35	D	2.16	3.40	100%	58.12%
Val36	D	2.53	2.34	99.99%	100%
Gly37	D	5.51	3.13	8.5%	43.75%
Gly38	D	7.00	3.75	2.02%	13.30%
Val39	D	6.09	2.33	12.92%	100%

*The average distance over 500 ns. ** % of frames with distance between EGCG and specific residues within 0.35 Å

Table S5. *Distances between EC and specific hotspots residues over 500 ns simulation.*

Pockets	Residues	Chains	Distance (Å)*		Distance within 0.35 Å**	
			Md1	Md2	Md1	Md2
P0	Val18	B	4.76	4.67	33.65%	43.13%
	Ala21	B	9.28	8.56	0.27%	0.51%
	Glu22	B	8.10	6.89	0.29%	1.14%
	Asp23	B	4.90	7.04	43.62%	43.68%
	Val24	B	3.89	7.21	53.08%	47.14%
	Gly25	B	6.94	9.50	0.01%	0.14%
	Ser26	B	5.18	7.90	6.5%	0.072%
	Asn27	B	4.19	7.16	34.2%	0.12%
	Phe20	C	2.80	3.26	94.86%	63.25%
	Ala21	C	2.51	3.07	98.45%	79.56%
	Glu22	C	1.96	3.78	93.70%	34.70%
	Gly25	C	6.23	9.15	6.66%	0.51%
	Ser26	C	4.35	6.95	17.55%	2.55%
	Asn27	C	2.39	2.89	99.97%	82.97%
	Lys28	C	2.28	1.84	86.91%	99.91%
	Gly29	C	5.06	2.96	8.85%	93.49%
	Ala30	C	6.49	2.76	0.30%	96.14%
	Ile31	C	7.40	3.46	0.03%	54.19%
	Val40	D	3.31	2.50	76.50%	96.53%
	Ile41	D	0.97	0.97	100%	100%
	Ala42	D	1.08	1.08	100%	100%
P1	Phe20	A	2.43	2.56	99.03%	96.73%
	Ala21	A	4.99	4.67	4.16%	38.60%
	Glu22	A	4.98	4.89	6.42%	47.71%
	Gly25	A	7.88	7.50	0.02%	0.44%
	Ser26	A	8.05	7.77	0.27%	2.11%
	Asn27	A	2.13	5.17	100%	7.18%
	Lys28	A	2.71	3.19	98.64%	64.47%
	Gly29	A	2.91	2.68	95.29%	90.48%
	Ala30	A	2.52	2.93	95.25%	90.67%
	Ile31	A	2.32	2.51	99.85%	96.87%
	Val40	B	3.40	3.29	68.82%	61.17%
	Ile41	B	2.94	2.62	76.56%	96.69%
	Ala42	B	1.08	2.52	100%	79.23%

	Val18	D	10.22	7.71	0%	3.70%
	Ala21	D	10.15	10.6	0.16%	0.04%
	Glu22	D	10.2	8.75	0.59%	0.51%
	Asp23	D	9.23	7.82	15.24%	1.89%
	Val24	D	7.40	8.50	14.52%	2.29%
	Gly25	D	6.84	7.93	1.27%	1.13%
	Ser26	D	5.65	5.45	27%	28.66%
	Asn27	D	9.05	8.03	0%	2.81%
P2	Leu17	A	4.30	4.79	49.84%	23.78%
	Val18	A	4.70	6.08	20.49%	0.026%
	Phe19	A	2.61	3.01	97.43%	91.43%
	Ile32	A	5.09	3.10	36.91%	89.35%
	Leu34	A	3.88	2.91	49.82%	86.41%
	Met35	B	3.11	3.35	80.86%	86.41%
	Val36	B	7.52	7.12	20.13%	0.012%
	Gly37	B	9.96	5.30	0.1%	24.01%
	Val39	B	9.59	4.95	0.14%	46.65%
	Leu17	C	6.47	3.41	17.48%	68.26%
	Val18	C	4.22	3.49	23.14%	61.50%
	Phe19	C	2.89	2.96	89.77%	88.25%
	Ile32	C	2.47	2.95	91.27%	83.76%
	Gly33	C	5.04	3.12	53.25%	75.62%
	Leu34	C	4.63	2.61	54.52%	98.13%
	Val36	C	8.96	5.08	5.55%	47.35%
D	Met35	D	7.28	6.65	39.88%	0.002%
	Val36	D	4.19	5.08	42.47%	47.35%
	Gly37	D	5.19	5.40	20.71%	41.40%
	Gly38	D	5.26	4.74	38.78%	42.43%
	Val39	D	7.91	7.90	0.008%	0%

*The average distance over 500 ns. ** % of frames with distance between EGCG and specific residues within 0.35 Å

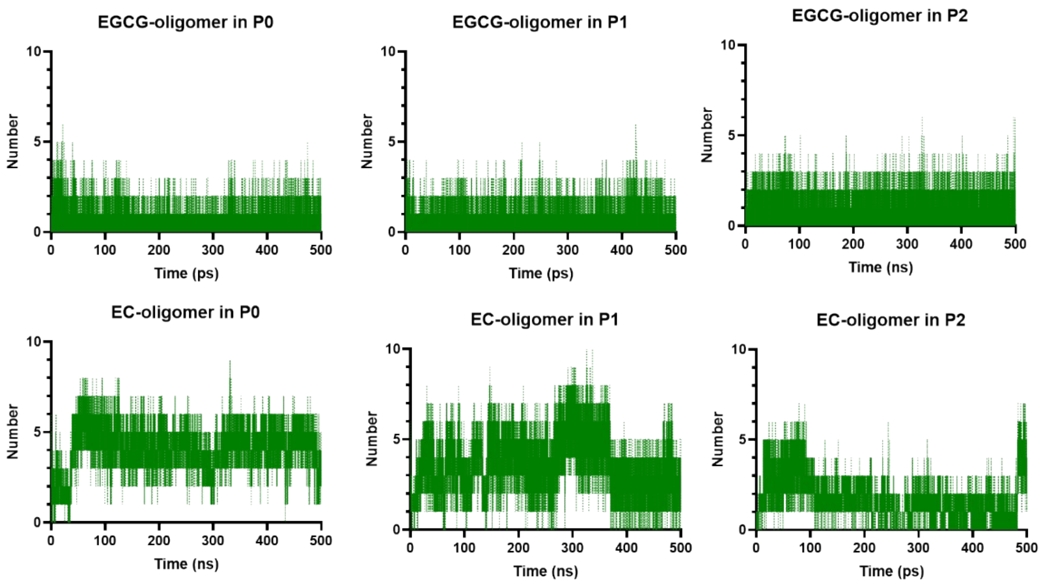

Figure S3. Number of H-bonds formed between EGCG or EC and the oligomer calculated over 500 ns for md1.

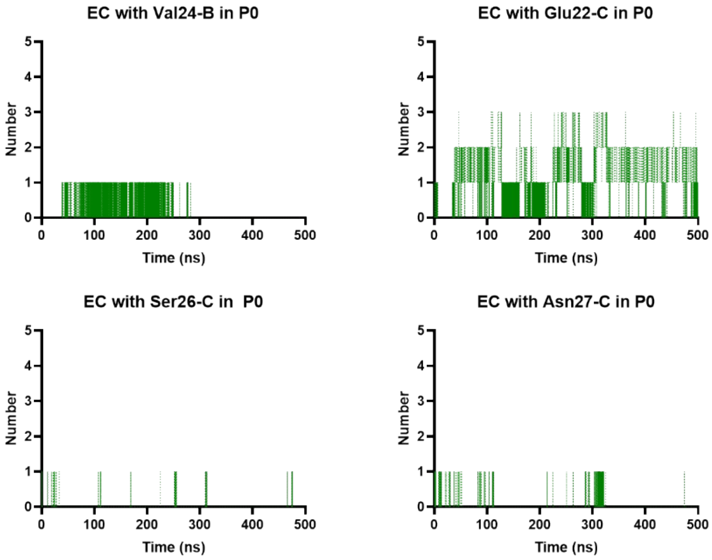

Figure S4. Prevalent H-bonds in P0 for EC-remodelled oligomer calculated over 500 ns for md1.

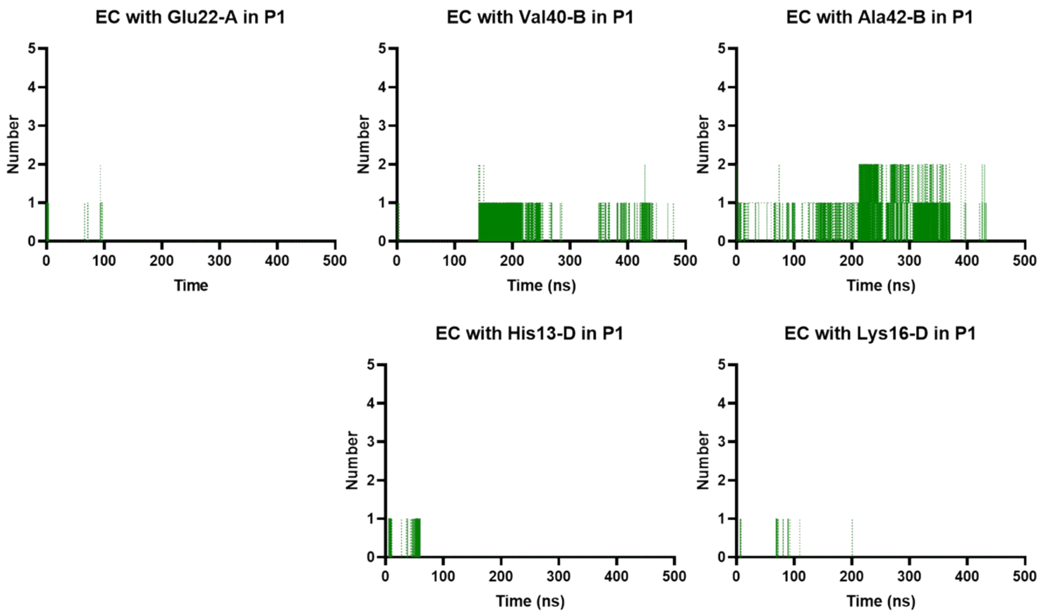

Figure S5. Prevalent H-bonds in P1 for EC-remodelled oligomer calculated over 500 ns for md1.

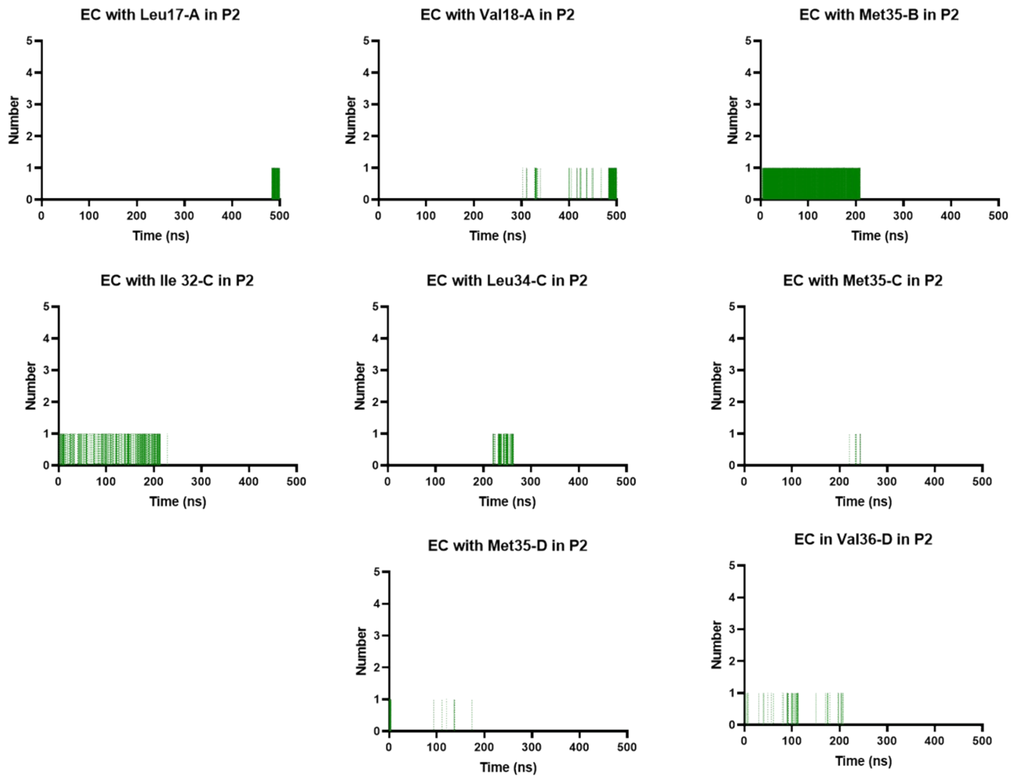

Figure S6. Prevalent H-bonds in P2 for EC-remodelled oligomer calculated over 500 ns for md1.

Table S6. Percentage of H-bond occupancy between EGCG or EC and a specific residues in each system with 500.000 coordinate snapshots.

Hotspots		***Residues***	***with EGCG***		***with EC***	
			md1	md2	md1	md2
P0	Chain B	Ala21-B	0.28%	0.62%	0.08%	0.02%
		Glu22-B	3.20%	0.36%	0.16%	0.53%
		Asp23-B	2.07%	-	-	-
		Val24-B	0.73%	-	25.3%	0.55%
	Chain C	Ser26-C	0.3%	1.9%	0.90%	0.84%
		Glu22-C	-	0.06%	81.3%	24.2%
		Asn27-C	0.11%	0.71%	2.7.%	0.38%
P1		Glu22-A	-	1.22%	0.86%	9%
	Chain A	Asn27-A	0.11%	0.44%	-	-
		Lys28-A	0.15%	1.9%	-	-
		Val40-B	-	-	10.3%	-
		Ala42-B	-	-	54.4%	48%
	Chain B	Ile41-B	1.16%	-	-	-
	Chain D	His13-D	-	-	0.42%	-
		His14-D	3.28%	0.2%	0.02%	0.03%
		Gln15-D	5.50%	0.17%	-	-
		Lys16-D	3.4%	0.45%	0.07%	-
		Glu22-D	0.10%	1.1%	-	-
		Val24-D	0.08%	0.99%	-	-
		Lys28-D	5.50%	0.26%	-	-
P2		Leu17-A	-	-	-	-
		Val18-A	-	-	2.7%	-
		Met35-B	-	-	16.9%	-
		Lys16-C	2.8%	-	-	-
		Leu17-C	1.96%	-	-	-
		Ile31-C	0.06%	0.97%	-	-
		Ile32-C	9.28%	3.35%	41.3%	3.9%
		Met35-C	14.24%	-	0.16%	-
		Leu34-D	2.2%	-	-	-
		Met35-D	4.93%	-	0.18%	-
		Val36-D	0.32%	3.15%	0.11%	-
